# Long-term outcomes of the largest (29) Epic Supra aortic valve bioprosthesis: comparing recommended with upsizing implantation

**DOI:** 10.1093/icvts/ivaf050

**Published:** 2024-03-05

**Authors:** Murat Mukharyamov, Hristo Kirov, Maria Golovina, Tulio Caldonazo, Torsten Doenst

**Affiliations:** Department of Cardiothoracic Surgery, Jena University Hospital, Friedrich Schiller University of Jena, Jena, Germany; Department of Cardiothoracic Surgery, Jena University Hospital, Friedrich Schiller University of Jena, Jena, Germany; Department of Cardiothoracic Surgery, Jena University Hospital, Friedrich Schiller University of Jena, Jena, Germany; Department of Cardiothoracic Surgery, Jena University Hospital, Friedrich Schiller University of Jena, Jena, Germany; Department of Cardiothoracic Surgery, Jena University Hospital, Friedrich Schiller University of Jena, Jena, Germany

**Keywords:** cardiac surgery, aortic valve replacement, biological prostheses, sizing

## Abstract

**OBJECTIVES:**

To investigate outcomes of aortic valve replacement with the largest available Epic Supra bioprosthesis (size 29).

**METHODS:**

We reviewed all patients who received an Epic Supra bioprosthesis between 2011 and 2023 and selected all 29 size prostheses that were implanted either into patients with an aortic annulus of at least 29 mm (sizing as recommended by the manufacturer) or into patients with smaller annuli (upsizing). Short- and long-term results were assessed. Propensity score matching was employed to improve comparability between groups. Kaplan–Meier and log-rank tests were performed to compare survival.

**RESULTS:**

Epic Supra bioprostheses were implanted into 1845 patients between 2011 and 2023. Mean age was 69.7 (11.2) years and 79.2% were male. Size 29 was implanted in 360 patients (mean age 68 (8.9) years), and 97.2% were male. EuroScore II was 5.1 (1.3; 6.6). One quarter of cases were performed via parasternal minithoracotomy. Mortality at 30 days was 2.8%, with no significant differences. Need for re-exploration was 3.3% and permanent pacemaker implantation was 4.7% (no statistically significant differences). Longest follow-up was 10 years with a mean of 50 (41) months. Mean prosthetic pressure gradients were 11.3 (16.7) mmHg and aortic valve reoperations/interventions were required in 5 patients (1.4%), all due to infective endocarditis.

**CONCLUSIONS:**

This is the largest experience of Epic Supra 29 implantation worldwide. Our results illustrate exemplary clinical and haemodynamic performance. In addition, the 29 size prosthesis can safely be implanted into patients with smaller than 29 mm annulus, which may improve future valve-in-valve options.

## GRAPHICAL ABSTRACT

**Figure:**
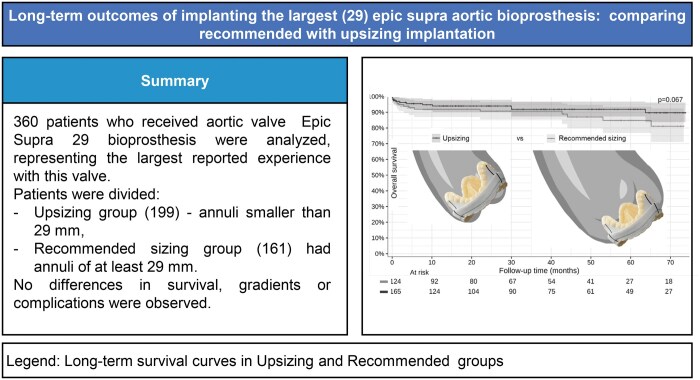

## INTRODUCTION

Aortic valve bioprostheses are provided in a wide range of sizes (classically labelled size 19 to 29). In general, the largest available prostheses are the ones least often implanted [1]. Large prostheses are generally associated with lower residual pressure gradients [2, 3] and the notion has been entertained that the largest prostheses are not even required for most patients. Thus, evidence for the largest bioprostheses is scarce.

We demonstrated that the implantation of a larger aortic valve prostheses (if technically feasible) into the same patient, known as ‘upsizing’, has the potential to improve haemodynamic performance [4]. Specifically, if the aortic root allows a replica sizer to fit which is bigger than the associated barrel sizer (e.g. a 23 replica sizer may fit the root but only a 21 barrel sizer fits the annulus). The larger prosthesis implanted (upsized) may then generate lower gradients [4, 5]. Yet, in patients receiving large prostheses, the anatomic dimensions may already suffice to generate good haemodynamics.

We therefore assessed our experience of implanting the largest available Epic Supra-aortic bioprosthesis into patients with anatomic dimensions that either did (i.e. the annulus was smaller than 29 mm) or did not allow upsizing (i.e. the annulus was 29 mm or larger) and compared outcomes including haemodynamic performance.

## MATERIALS AND METHODS

Data from hospital records were extracted for patients who received an Epic Supra as isolated aortic valve replacement or combined procedures, regardless of valve disease aetiology between 2011 and 2023. We selected all patients who received a 29-size prosthesis and divided them into 2 groups: those implanted into an aortic annulus where the barrel end of the sizer indicated an annular size of at least 29 mm (recommended sizing group) and those implanted into an aortic annulus where the 29 barrel end of the sizer did not pass the annulus (upsizing group). The primary end-point of the study was long-term survival, with secondary end-points including the rate of reoperations, immediate and long-term pressure gradients, and the need for permanent pacemaker implantation.

Isolated or combined aortic valve replacement surgeries were performed either through a median sternotomy with central cannulation or using a minimally invasive approach via a parasternal approach. The mini group also contained double and triple valve procedures. For the parasternal approach, cardiopulmonary bypass (CPB) was established using percutaneous groin cannulation. A 4–5 cm right parasternal incision was made over the second intercostal space, and the pleural space was opened. The right internal mammary artery and vein were ligated and transected, and the third rib was detached at the sternal joint. CPB was initiated, and the pericardium was opened. A left atrial vent and a cardioplegia cannula were placed, and the aorta was cross-clamped using the Cygnet clamp. Bretschneider's solution was administered as a single-shot antegrade cardioplegia. At the end of the procedure, the pericardium was closed, the rib was reattached with a 1–0 vicryl suture, and the wound was closed in layers. CPB was terminated, and the Proglide system was used to close the arterial puncture site. In cases where intervention on the atrioventricular valves was required, the skin incision was shifted one intercostal space lower and more laterally. Access to the mitral valve was achieved through the interatrial groove, while the tricuspid valve was approached via the right atrial wall.

The measurement of the annulus dimensions was performed by identifying the largest barrel sizer that could pass through the annulus (intra-annular sizing). For example, if the largest barrel sizer that could be introduced was size 25, the annulus was classified as 25/26 mm in diameter, as the next size (27) could not fit. The sizer was then flipped to test the replica end of the same size, which was used to evaluate the fit of the prosthesis for supra-annular placement within the aortic root. If the replica was deemed suitable by the surgeon, it aligned with the manufacturer’s recommendation to implant a prosthesis of that size (http://www.accessdata.fda.gov/cdrh_docs/pdf10/P100029c.pdf). In practice, however, larger replica sizers were often tested to evaluate their compatibility with the root anatomy. If a larger replica sizer could be introduced into the root (frequently by tilting) and its positioning within the root including the alignment of the commissures, annular sections, and coronary ostia was assessed as acceptable by the surgeon, a prosthesis 1 or 2 sizes larger was selected for implantation. This process, when deemed appropriate by the operating surgeon, termed ‘upsizing’ in this study, involved choosing a prosthesis based on the fit of a replica. Both the measured annulus dimensions and the implanted valve sizes are consistently recorded in our surgical notes. The prostheses have been implanted using sequential U-shaped sutures of polyfilament thread with synthetic pledgets suturing through the annulus from the ventricular side towards the sinuses.

We assessed perioperative outcomes, including 30-day mortality, re-thoracotomies, the need for permanent pacemaker implantation, and postoperative pressure gradients over the prosthesis. Additionally, long-term outcomes were assessed, focusing on actuarial survival and pressure gradients over the prostheses. Propensity score matching (PSM) was employed to minimize selection bias and ensure comparability between the 2 sizing groups with the formation of 75 patient pairs.

Statistical analysis and data visualization were conducted using the R 4.3.1 statistical computing environment (R Foundation for Statistical Computing, Vienna, Austria). PSM was performed using the MatchIt 4.5.5 package. To assess covariate balance (potential confounders), standardized mean difference was used. Logistic regression was employed for PSM, with untransformed covariates included as predictors. The nearest neighbour method was used for 1 to 1 PSM with caliper 0.1. The normality of continuous variables was assessed with Shapiro–Wilk test and visual inspection of qq-plots in each full cohort group. For comparing full cohort groups regarding quantitative variables, the Mann–Whitney test was utilized, while for comparing groups in terms of categorical variables, the Pearson's chi-squared test was applied. Survival analysis employed the Kaplan–Meier estimator, log-rank test, and univariable Cox proportional hazards models to estimate hazard ratios; simplified person-time method was used to estimate the follow-up rate. For aortic valve reintervention analysis, incidence rates with corresponding 95% CIs were estimated. For comparing matched groups regarding quantitative variables, the Wilcoxon signed-rank test was used, while for comparing groups in terms of binary and ordered variables, the McNemar's and McNemar–Bowker tests were applied. Quantile regression model with time included as covariate after restricted cubic spline transformation was used to compare mean gradient at follow-up. For survival analysis in matched groups, the Kaplan–Meier estimator and likelihood ratio test for clustered Cox proportional hazard model were used. Survival curves were truncated at time point when less than 10% of the original sample size remained is at risk.

## RESULTS

Out of 1845 screened patients who received an Epic Supra bioprosthesis between 2011 and 2023, 360 with a size 29 valve were included in the analysis. These patients were distributed into the recommended sizing group (*n* = 161) and the upsizing group (*n* = 199). Table 1 shows baseline demographic data. Mean age was 67.7 (8.9) years and 97% of patients were male with a mean body mass index of 28.6 (5.2). The majority of patients were in New York Heart Association class II or III. Median ejection fraction was 55% (43, 63). One third of patients suffered from concomitant coronary artery disease, 17% from concomitant mitral and/or tricuspid valve disease, and 7% of patients were operated with the diagnosis of acute infective endocarditis. Median EuroScore II was 5.1 (1.3; 6.6). The Society of Thoracic Surgery (STS) score was 1.72 (0.99; 3.01) which, however, does not account for concomitant aortic surgery that was performed in over 20% of patients. There was no relevant difference between the recommended and the upsizing groups.

**Table 1:** Baseline demographic and clinical data

Characteristic	Overall (*n* = 360)	Recommended sizing (*n* = 161)	Upsizing (*n* = 199)	*P*-value
Age, mean (SD)	67.7 (8.9)	67.2 (9.6)	68.0 (8.4)	0.618
Male gender, %	350 (97.2%)	158 (98.1%)	192 (96.5%)	0.531
BMI, mean (SD)	28.6 (5.2)	28.0 (5.5)	29.1 (5.0)	0.027
BSA, mean (SD)	2.03 ± 0.18	2.02 (0.17)	2.04 (0.18)	0.056
Diabetes mellitus, %	65 (18.1%)	19 (11.8%)	46 (23.2%)	0.008
Heart failure functional class NYHA (%)				0.016
0	23 (6.4%)	16 (9.9%)	7 (3.5%)	
I	34 (9.4%)	11 (6.8%)	23 (11.6%)	
II	104 (28.9%)	40 (24.8%)	64 (32.2%)	
III	175 (48.6%)	86 (53.4%)	89 (44.7%)	
IV	24 (6.7%)	8 (5.0%)	16 (8.0%)	
Infective endocarditis	24 (6.7%)	11 (6.8%)	13 (6.5%)	0.912
Arterial hypertension, %	85 (23.6%)	47 (29.2%)	38 (19.1%)	0.034
Pulmonary hypertension, %	76/296 (25.7%)	36 (30.3%)	40 (22.6%)	0.18
Coronary artery disease, %	138 (38.3%)	52 (32.3%)	86 (43.2%)	0.044
Atrial fibrillation, %	22 (6.1%)	15 (9.3%)	7 (3.5%)	0.039
Peripheral atherosclerosis, %	27 (8.7%)	15(11.6%)	12 (6.6%)	0.182
Chronic kidneys disease, %	5 (1.4%)	3 (1.9%)	2 (1.0%)	0.811
Left ventricular ejection fraction, median (1st, 3rd quartiles)	55 (43, 63)	55 (41, 62)	55 (44, 64)	0.347
Valve anatomy				>0.999
Tricuspid, %	216 (60.3%)	96 (60.4%)	120 (60.3%)	
Bicuspid, %	142 (39.7%)	63 (39.6%)	79 (39.7%)	
EuroScore II, median (1st , 3rd quartiles)	5.1 (1.3; 6.6)	5.2 (1.3; 6.7)	5.0 (0.9; 6.6)	0.374
STS risk score, median (1st , 3rd quartiles)	1.72 (0.99, 3.01)	1.67 (0.99, 2.72)	1.74 (1.00, 3.21)	0.531

BMI, body mass index; BSA, body surface area; NYHA, New York Heart Association; SD, standard deviation; STS, Society of Thoracic Surgery.

Table 2 shows the operative data. There was more concomitant coronary artery bypass grafting (CABG) in the upsizing group and more supracoronary aortic replacement in the recommended sizing group. Three quarters of patients received the procedure through median sternotomy and one quarter through minithoracotomy.

**Table 2:** Operative data

Characteristic	Overall (*n* = 360)	Recommended sizing (*n* = 161)	Upsizing (*n* = 199)	*P*-value
Median sternotomy, %	262 (72.8%)	122 (75.8%)	140 (70.4%)	0.303
Parasternal minithoracotomy, %	98 (27.2%)	39 (24.2%)	59 (29.6%)	
Cardiopulmonary bypass time (min), median (1st, 3rd quartiles)	105 (82, 128)	104 (82, 133)	106 (83, 126.5)	0.879
Aortic cross-clamp time (min), median (1st, 3rd quartiles)	67 (53, 83.3)	69 (52, 87)	66 (53, 82.5)	0.435
CABG, %	67 (18.6%)	18 (11.2%)	49 (24.6%)	0.002
MVR(r), %	54 (15.0%)	26 (16.1%)	28 (14.1%)	0.689
TVR, %	54 (15.0%)	26 (16.1%)	28 (14.1%)	0.689
Bentall procedure, %	20 (5.6%)	10 (6.2%)	10 (5.0%)	0.797
Supracoronary aortic replacement, %	58 (16.1%)	35 (21.7%)	23 (11.6%)	0.014

min, minute; CABG, coronary artery bypass grafting; MVR(r), mitral valve repair (replacement); TVR, tricuspid valve repair.

There were no differences in the sizing or selection of valve prostheses compared to the full sternotomy approach. Among the 98 patients who underwent parasternal minithoracotomy, 59 (60.2%) received an upsized implantation. The surgical approach (median sternotomy or parasternal minithoracotomy) did not impact the feasibility of upsized implantation, as demonstrated by similar rates of upsizing in both groups.

Table 3 shows short- and long-term outcomes with the longest follow-up being 10 years and 62.9% 5-year follow-up rate. Mortality at 30 days was 2.8% in the general cohort with no difference between the subgroups. The percentage of redo thoracotomies for bleeding was 3.3%. It was higher in the upsizing group, but the difference was not statistically significant (*P* = 0.27). The requirement for permanent pacemaker implantation was 4.7%, again with no difference between subgroups. Postoperative mean gradient at discharge was 9 (7, 12) mmHg at discharge and around 11 mmHg at latest follow-up. There were no differences between the recommended sizing and the upsizing groups. Valve-related reoperations or reinterventions by catheter during follow-up was required in 5 patients (1.4%, 2 in the recommended sizing group and 3 in the upsizing group, *P* = 0.999) occurring at an average time of 47.4 (41.3) months after the index operation. The reason for reintervention was prosthetic endocarditis in all 5 cases. There were 4 cases (1.1%) with paravalavular leaks showing trace to mild regurgitation (2 in each group), which was present at discharge and did not change during follow-up.

**Table 3:** Perioperative and long-term outcomes

Characteristic	Overall (*n* = 360)	Recommended sizing (*n* = 161)	Upsizing (*n* = 199)	*P*-value
30-day mortality	2.8%	2.5%	3.0%	>0.999
Rethoracotomy for bleeding	3.3%	1.9%	4.5%	0.27
Pacemaker implantation	4.7%	5.6%	4.0%	0.654
Mean gradient at discharge (mmHg), median (1st, 3rd quartiles)	9 (7, 12)	10 (8, 13)	9 (7, 11)	0.156
Observed at last time point, median (1st, 3rd quartiles)	9 (7, 11)	9 (7, 11)	8 (7, 11)	0.276
Estimated median (1st, 3rd quartiles) at 1 year after surgery	9 (9, 11)	9 (9, 12)	9 (8, 11)	0.318
Estimated median (1st, 3rd quartiles) at 3 year after surgery	9 (7, 11)	9 (8, 12)	9 (7, 11)	0.392
Estimated median (1st, 3rd quartiles) at 5 year after surgery	9 (7, 12)	10 (8, 13)	9 (7, 12)	0.102
Aortic valve reintervention (number of cases/PTY/incidence rate (per 100 PTY) [95% CI])	5/920 0.54 [0.18; 1.27]	2/389 0.51 [0.06; 1.86]	3/531 0.56 [0.12l 1.65]	0.917

mmHg, millimeters of mercury; PTY, patient/years.

Tables S1–S4 show the demographic, operative and outcomes data after PSM. Risk adjustment did not change any of the findings in the general cohort. Table S2 shows results of covariates balance assessment in full cohort and after PSM procedure. Figure S1 shows overlap of distribution of estimated propensity scores across compared groups in full and matched cohorts.

**Figure 1:**
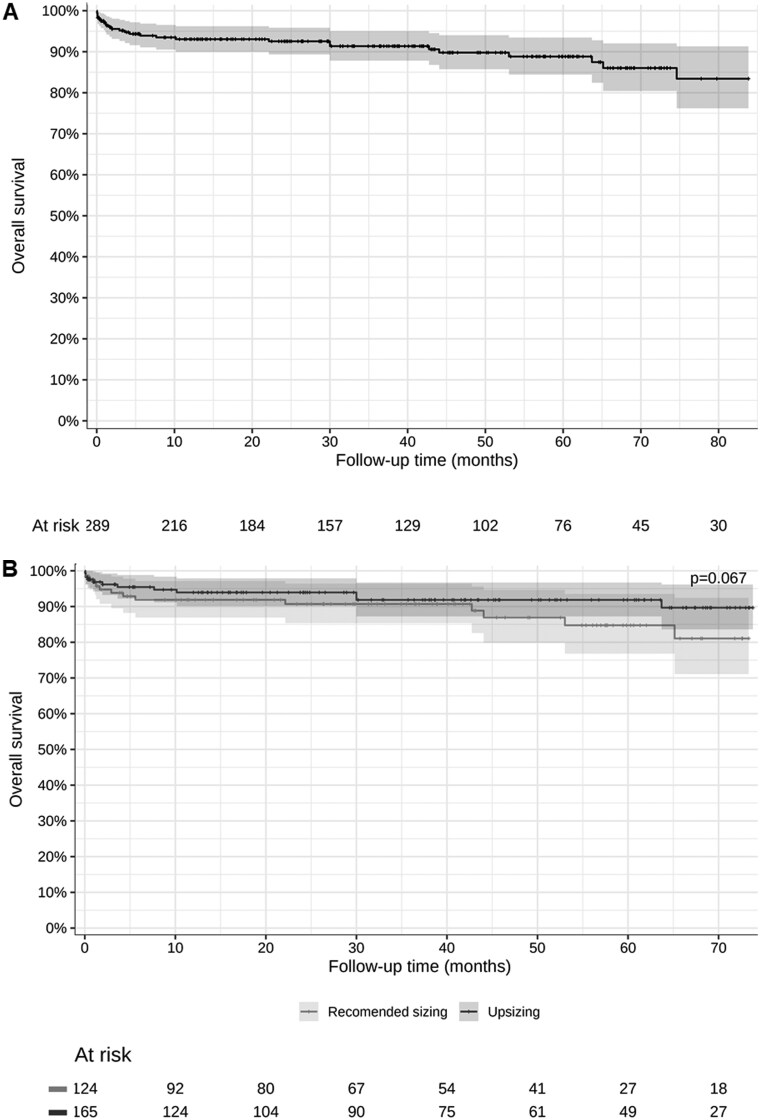
(A) Long-term survival in the general study cohort. (B) Long-term survival curves in recommended and upsizing groups. Figure 1 shows survival curves of the full cohort (A) and of the recommended sizing and the upsizing groups (B). Figure 1B illustrates a numerically better survival in the upsizing group. However, the difference was not statistically significant.

Figure 1 shows actuarial survival curves for the full cohort (A) and the recommended sizing and upsizing groups (B), with long-term survival rates of 84.1% in the overall cohort, 80.8% in the recommended sizing group, and 90.2% in the upsizing group (HR = 0.51; 95% CI: 0.24–1.06). Figure 1B illustrates a numerically better survival in the upsizing group. However, the difference was not statistically significant (*P* = 0.067).

Data from the PSM are displayed in Tables S1–S4 and Fig. S2. None of the outcomes was significantly altered through this risk adjustment, including the survival outcomes.

## DISCUSSION

We here present the largest experience of Epic Supra 29 implantations worldwide. Our results illustrate exemplary clinical and haemodynamic performance. In addition, the 29 size prosthesis can safely be implanted into patients with smaller than 29 mm annulus, which may improve future valve-in-valve options.

Our data show very good and comparable haemodynamic performance to other large valve prostheses [6–8] and was associated with a very low risk of reinterventions on the aortic valve of 0.54 [95% CI: 0.18–1.27] per patient-year (5 patients out of a total cohort of 360 observations). Importantly, with 62.9% 5-year follow-up rate, all reinterventions were associated with infective endocarditis. There was no individual valve failure or early structural degeneration. Although our observation period is still limited, the current results support the unsurpassed, good long-term outcomes of the Epic porcine valve platform [1, 9, 10].

Despite these results, the overall use of the Epic valve worldwide has been limited. The largest published series of patients having received Epic Supra valve implantations in the aortic position stems from the U.S. Medicare population during the period of 2008–2019 [1]. It contained 11 685 patients, (4.8%) of an overall of 244 420 patients having received an aortic valve replacement during that time. Of these Epic Supra prostheses, 11.5% were size 19 mm, 29.9% size 21, 34% size 23, and 19% size 25. The absolute number of patients with a 27 prosthesis was 652 (5.6%) and there was no patient with a size 29. The latter is due to the fact that there is no FDA-approval for the 29 size Epic Supra in the United States, which again is due to the lack of sufficient information on this type of valve. It is interesting that the 27 size Epic Supra was implanted in only 5.6% of the Medicare population, while in our patient population almost half (47.5%) of patients received a 27 or a 29 size valve. The difference is not easily explained because anatomic differences between German and US American patients are unlikely. They may be due to the fact that it has been our rationale to implant the largest possible size even in patients with smaller anatomy, while until recently the general notion may have been that such valve sizes may not be required.

Since we have routinely assessed the aortic root with the replica sizer, we ended up implanting larger sizes than recommended in almost two thirds of patients [4]. In the current patient population, more than half of the cases had aortic annuli smaller than the barrel sizer for the 29 mm Epic Supra valve. While in smaller sizes, upsizing reduced pressure gradients [4], this was not the case the current series. Pressure gradients were not different in the upsizing group. While a direct assessment of the impact of upsizing cannot be done in this data set (because we would have to compare the upsized 29 valves to recommended sized 27 valves), the gradients of around 10 mmHg are comparable to other valves of larger size [6, 9, 11], including mechanical valves [12]. The latter have much larger actual opening areas than all biological prostheses, which suggests that the detected gradients may not primarily be determined by valve opening area anymore. This recognition lends support again to the above described notion of what is the actually required opening area.

However, modern patient life-time management strategies include the implantation of transcatheter valves into previously implanted prostheses [13]. Novel biological valve designs include the possibility to expand the implanted valve stent [14]. The nature of the Epic stent allows for fracturing it, during a valve-in-valve procedure [15]. In any case, implanting the largest stent size possible at the time of the index operation appears to be the best and possibly safest way to optimize future treatment options, including valve-in-valve procedures, which currently suffer substantially from inferior haemodynamics in the long run [16].

Another important recognition is the fact that the use of our upsizing strategy did not show any significant clinical or haemodynamic difference, including during follow-up. In other words, the 29-size prosthesis performed equally well, whether implanted into an annulus of at least 29 mm or in a smaller annulus. Upsizing of the surgically implanted valve in our study was not associated with increased risks of mortality or pacemaker implantation, which may be quite the opposite in the case of upsizing implantation of transcatheter or so-called sutureless valves [17]. To the contrary, observed to expected mortality was substantially below one with respect to Euroscore II and around one compared to the Society of Thoracic Surgery (STS) score, which did not take the aortic procedures that were performed in every fifth patient into account and which was revised at least once during the observation period, which results in an underestimation of risk for a retrospective observation period.

The current ‘competition’ of TAVI (transcatheter aortic valve implantation) vs SAVR (surgical aortic valve replacement) may make our rates of pacemaker implantation (4.0% and 5.6%) appear high, considering that individual randomized cohorts already show pacemaker rates as low as 5% [18]. In randomized controlled trials (specifically the one cited), TAVI procedures are performed in highly selected patients, excluding pathological conditions such as bicuspid aortic valve or endocarditis. In contrast, our cohort included a bicuspid valve frequency of 39.7%, an infective endocarditis frequency of 6.7%, and combined interventions on atrioventricular valves in 15% of cases. All these factors increase the risk of requiring permanent pacemaker implantation. The incidence of permanent pacemaker implantation in our study among patients undergoing isolated aortic valve replacement, excluding those with endocarditis, bicuspid aortic valves, and concomitant procedures (other than CABG), was 3.8%. At the same time, real-world data, specifically the results from the German Aortic Valve Registry (GARY) [19], show a pacemaker implantation risk of 4.6% for isolated surgical valve replacement and 18.1% for TAVI. Similarly, the Italian OBSERVANT registry [20] reported pacemaker implantation rates of 7.3% for SAVR and 18.5% for TAVI, respectively. From this ‘daily clinical practice’ perspective, our results probably reflect the true risks for the patient population included in our study and appear to be rather on the low side.

Finally, patients with aortic annuli in a size range for receiving a 29 mm valve often require root or ascending aorta replacement. In our experience, the incidence of complete aortic root replacement (Bentall procedure) or supracoronary aortic replacement (Table 2) was significantly higher (*P* = 0.014) in the recommended sizing group, with 45 cases (27.9%), compared to the upsizing group, which had 33 cases (16.6%), which may be explained by the notion that patients with aneurysmatic aortas are likely to also have larger annuli and upsizing is then not possible anymore. It is our rationale to perform supracoronary replacement in cases with dilated aortas if the root remains below 45 mm in diameter, based on the recognition that long-term outcomes in such cases do not differ from full root replacements [21], while reoperations following Bentall procedures carry substantial risk [22, 23].

The limitations of the present study include its retrospective nature, single-centre design, and the significant heterogeneity of the study cohort, including different aetiologies of aortic valve disease and combined procedures.

## CONCLUSIONS

The Epic Supra 29 demonstrates good clinical and haemodynamic results post-implantation. Transprosthetic gradients remained low over the observation periods of up to 10 years, and reoperation rates were very low during follow-up and were not related to structural valve deterioration. Implanting the size 29 prosthesis into a narrower aortic annulus is safe and this upsizing technique can provide an additional tool to improve future valve-in-valve options.

## Supplementary Material

ivaf050_Supplementary_Data

## Data Availability

The data underlying this article will be shared on reasonable request to the corresponding author.

## Notes

Presented at the EACTS/STS/LACES Latin America Cardiovascular Surgery Conference, Buenos Aires, Argentina, December, 2024.

## ABBREVIATIONS

CABG: Coronary artery bypass grafting
CPB: Cardiopulmonary bypass
PSM: Propensity score matching
STS: Society of Thoracic Surgery
TAVI: Transcatheter aortic valve implantation
