# Supplementary material for: Long-term outcomes of the largest (29) Epic Supra aortic valve bioprosthesis: comparing recommended with upsizing implantation
Source: Interdiscip Cardiovasc Thorac Surg. 2024 Mar 5;40(3):ivaf050. doi: 10.1093/icvts/ivaf050 (PMC11928935; doi:10.1093/icvts/ivaf050)
Supplement: ivaf050_Supplementary_Data [file ivaf050_supplementary_data.zip › Supplemental Material_20012024.docx]

**Supplemental Material**

Supplementary table 1. Baseline demographic and clinical data after PSM

| **Characteristic** | **Overall**  (n- 150) | **Recommended sizing**  (n- 75) | **Upsizing**  (n - 75) | **p-value** |
| --- | --- | --- | --- | --- |
| Age (mean (SD)) | 67.6 (9.0) | 67.3 (8.8) | 67.8 (9.2) | 0.915 |
| Male gender (%) | 46 (97.3%) | 73 (97.3%) | 73 (97.3%) | >0.999 |
| BMI (mean (SD)) | 28.9 (5.2) | 28.7 (5.9) | 29.1 (4.4) | 0.327 |
| Diabetes mellitus (%) | 24 (16.0%) | 11 (14.7%) | 13 (17.3%) | 0.789 |
| Heart failure functional class NYHA (%) | | | | 0.986 |
| 0 | 2 (1.3%) | 1 (1.3%) | 1 (1.3%) |  |
| I | 7 (4.7%) | 4 (5.3%) | 3 (4.0%) |  |
| II | 44 (29.3%) | 21 (28.0%) | 23 (30.7%) |  |
| III | 89 (59.3%) | 45 (60.0%) | 44 (58.7%) |  |
| IV | 8 (5.3%) | 4 (5.3%) | 4 (5.3%) |  |
| Endokarditis | 4 (2.7%) | 2 (2.7%) | 2 (2.7%) | 0.89 |
| Arterial hypertension (%) | 41 (27.3%) | 22 (29.3%) | 19 (25.3%) | 0.710 |
| Pulmonary hypertension (%) | 44 (29.3%) | 24 (32.0%) | 20 (26.7%) | 0.607 |
| Coronary artery disease (%) | 6 (37.3%) | 29 (38.7%) | 27 (36.0%) | 0.864 |
| Atrial fibrillation (%) | 4 (2.7%) | 2 (2.7%) | 2 (2.7%) | >0.999 |
| Peripheral atherosclerosis (%) | 3 (2.0%) | 2 (2.7%) | 1 (1.3%) | >0.999 |
| Chronic kidneys disease (%) | 1 (0.7%) | 0 (0.0%) | 1 (1.3%) | >0.999 |
| Left ventricular ejection fraction (median (1st, 3rd quartiles) | 55.0 (46.3, 62.0) | 56.0 (45.0, 62.0) | 55.0 (46.5, 63.5) | 0.54 |
| Valve anatomy (%) | | | | 0.864 |
| Tricuspid (%) | 86 (57.3%) | 44 (58.7%) | 42 (56.0%) |  |
| Bicuspid (%) | 64 (42.7%) | 31 (41.3%) | 33 (44.0%) |  |
| EuroScore II (median (1st, 3rd quartiles) | 3.0 (1.3; 7.3) | 3.0 (1.6; 8.8) | 1.9 (1.3; 7.3) | 0.281 |
| STS risk score (median (1st, 3rd quartiles) | 1.7 (1.1, 2.6) | 1.6 (1.1, 2.4) | 1.8 (1.1, 2.7) | 0.876 |

*BMI – body mass index, NYHA – New-York Heart Assosiation, SD- standard deviation, STS – society of thoracic surgery*

Supplementary table 2. Results of covariate balance assesment (standardized mean differrences, SMD’s) in full cohort and in mathched groups.

| **Characteristic** | Full cohort | Matched groups |
| --- | --- | --- |
| Age | 0.06 | 0.06 |
| Male gender | **-0.11** | 0.00 |
| BMI | **0.14** | 0.09 |
| Diabetes mellitus | **0.28** | 0.06 |
| NYHA 0 | -0.05 | 0.00 |
| NYHA I | **0.17** | -0.05 |
| NYHA II | **0.17** | 0.06 |
| NYHA III | **-0.30** | -0.03 |
| NYHA IV | 0.08 | 0.00 |
| Arterial hypertension | **-0.30** | -0.09 |
| Pulmonary hypertension | **-0.10** | **-0.12** |
| Coronary artery disease | **0.12** | -0.05 |
| Atrial fibrillation | -0.03 | 0.00 |
| Peripheral atherosclerosis | **0.11** | -0.06 |
| Chronic kidneys disease | -0.07 | **0.11** |
| STS | **-0.10** | -0.01 |
| Left ventricular ejection fraction | **0.14** | 0.09 |
| Bicuspid valve anatomy | 0.00 | 0.05 |

Supplementary table 3. Operative data after PSM

| Characteristic | Overall  (n- 150) | Recommended sizing  (n- 75) | Upsizing  (n - 75) | p-value |
| --- | --- | --- | --- | --- |
| Median sternotomy (%) | 110 (73.3%) | 57 (76.0%) | 53 (70.7%) | 0.54 |
| Parasternal minithoracotomy (%) | 40 (26.7%) | 18 (24.0%) | 22 (29.3%) |  |
| Cardiopulmonary bypass time (min) (median (1st, 3rd quartiles) | 106 (83, 134) | 106 (86, 139.5) | 106 (82, 125) | 0.393 |
| Aortic cross-clamp time (min) (median (1st, 3rd quartiles) | 68.5 (53, 84) | 73 (54, 92.5) | 64 (50.5, 76.5) | 0.006 |
| CABG (%) | 8 (18.7%) | 11 (14.7%) | 17 (22.7%) | 0.286 |
| MVR(r) (%) | 6 (10.7%) | 11 (14.7%) | 5 (6.7%) | 0.211 |
| TVR (%) | 9 (6.0%) | 7 (9.3%) | 2 (2.7%) | 0.182 |
| Bentall procedure (%) | 10 (6.7%) | 5 (6.7%) | 5 (6.7%) | >0.999 |
| Supracoronary aortic replacement (%) | 28 (18.7%) | 18 (24.0%) | 10 (13.3%) | 0.136 |

*min – minutes , CABG – coronary artery bypass grafting, MVR(r) – mitral valve repair(replacement), TVR - tricuspid valve repair*

Supplementary table 4. Immediate and long-term outcomes after PSM

| **Characteristic** | **Overall**  n = 150 | **Recommended sizing**  n = 75 | **Upsizing**  n = 75 | **p-value** |
| --- | --- | --- | --- | --- |
| 30-day mortality | 4 (2.7%) | 2 (2.7%) | 2 (2.7%) | >0.999 |
| Rethoracotomy for bleeding | 7 (4.7%) | 1 (1.3%) | 6 (8.0%) | 0.131 |
| Pacemaker implantation | 8 (5.3%) | 3 (4.0%) | 5 (6.7%) | 0.716 |
| Mean gradient at discharge (mmHG) (median (1st, 3rd quartiles) | 9 (8, 12) | 9 (8, 12) | 9 (7, 12.5) | 0.739 |
| Mean gradient long term (mmHg) (median (1st, 3rd quartiles) | 8 (7, 11) | 8 (7, 11) | 8.5 (7, 12) | 0.44 |
| Aortic valve re-intervention | – | – | – | – |

*mmHG – millimeters of mercury*

**Figure legends**

Supplementary Figure 1. Distribution of estimated propensity scores across compared groups in full and matched cohorts.

Supplementary Figure 2. Long-term survival curves after PSM
