# Supplementary figures and images for: Long-term outcomes of the largest (29) Epic Supra aortic valve bioprosthesis: comparing recommended with upsizing implantation
Source: Interdiscip Cardiovasc Thorac Surg. 2024 Mar 5;40(3):ivaf050. doi: 10.1093/icvts/ivaf050 (PMC11928935; doi:10.1093/icvts/ivaf050)

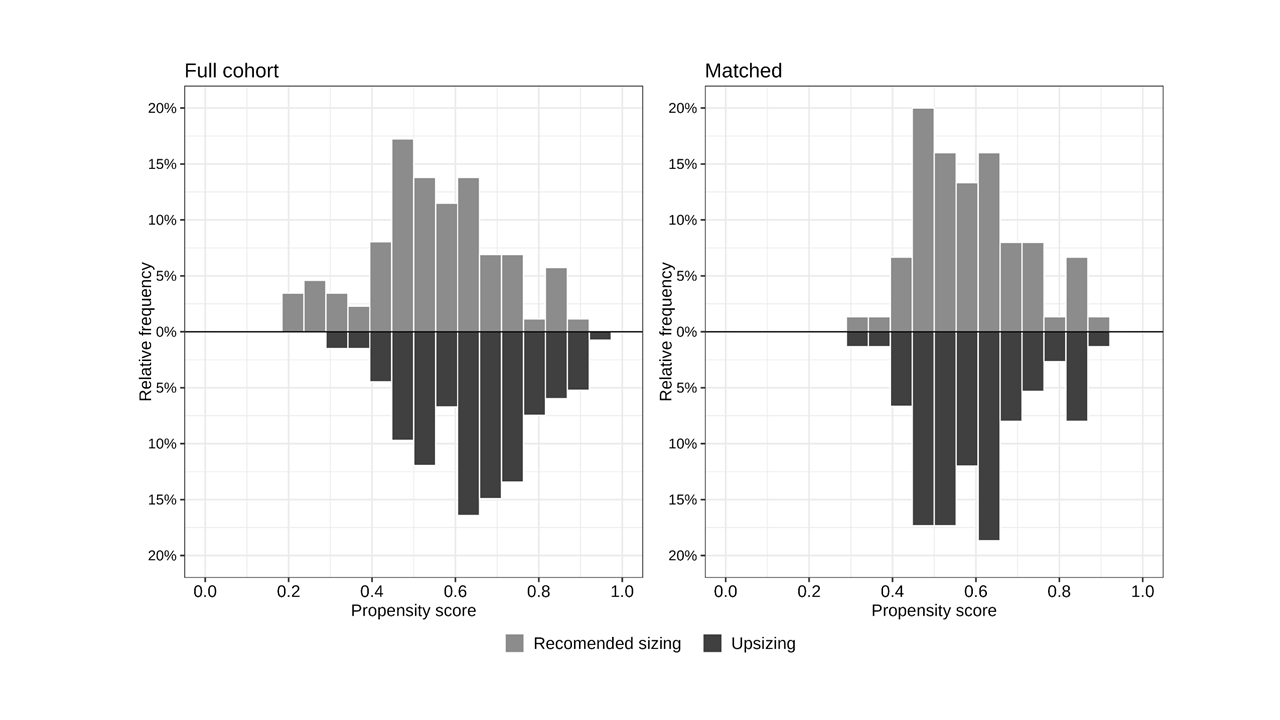

Supplement: ivaf050_Supplementary_Data [file ivaf050_supplementary_data.zip › SuppFig1.TIF]

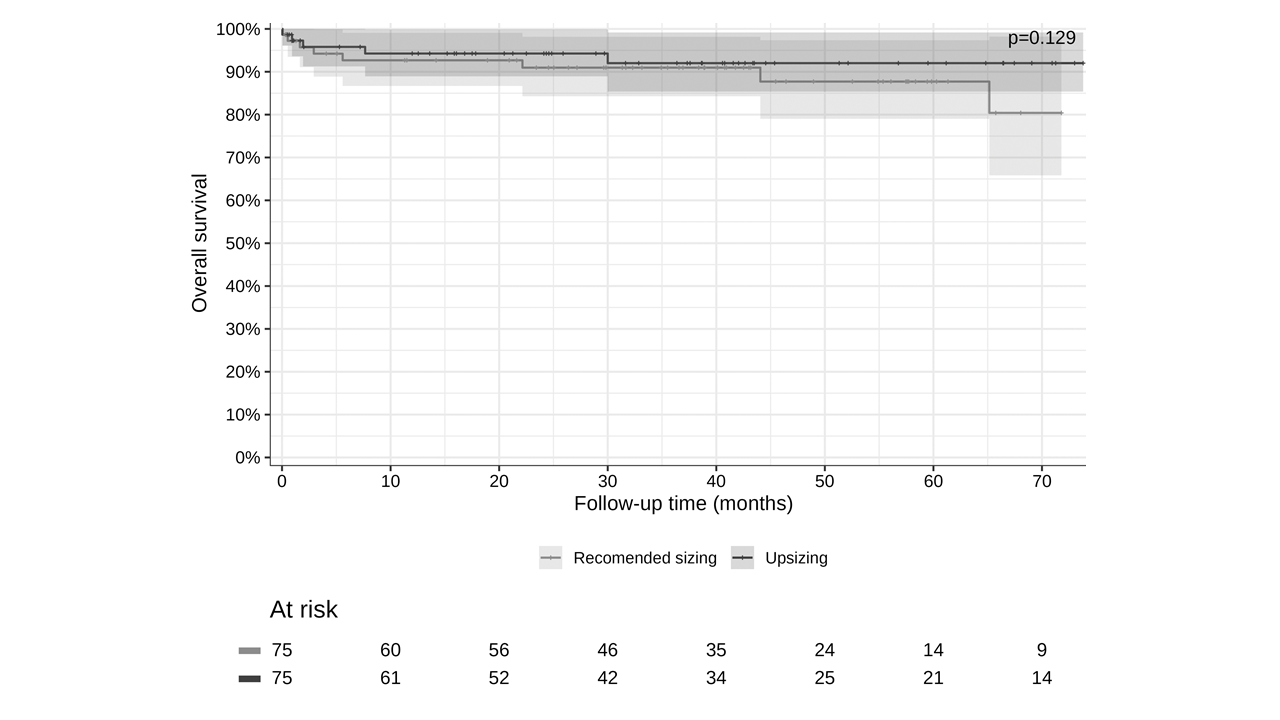

Supplement: ivaf050_Supplementary_Data [file ivaf050_supplementary_data.zip › SuppFig2.TIF]
